# Barriers and Solutions to Addressing Tobacco Dependence in Addiction Treatment Programs

**Published:** 2006

**Authors:** Douglas M. Ziedonis, Joseph Guydish, Jill Williams, Marc Steinberg, Jonathan Foulds

**Affiliations:** Douglas M. Ziedonis, M.D., M.P.H., is a professor and chair, Department of Psychiatry, University of Massachusetts Medical School, Worcester, Massachusetts. Joseph Guydish, Ph.D., M.P.H., is an assistant adjunct professor, Medicine and Health Policy, University of California, San Francisco, California. Jill Williams, M.D., is an associate professor, Department of Psychiatry, University of Medicine and Dentistry of New Jersey (UMDNJ) Robert Wood Johnson Medical School; and the UMDNJ School of Public Health Tobacco Dependence Program, New Brunswick, New Jersey. Marc Steinberg, Ph.D., is an assistant professor, Department of Psychiatry, UMDNJ Robert Wood Johnson Medical School, New Brunswick, New Jersey. Jonathan Foulds, Ph.D., is an associate professor and director, Tobacco Dependence Program, UMDNJ Robert Wood Johnson Medical School; and the UMDNJ School of Public Health Tobacco Dependence Program, New Brunswick, New Jersey

**Keywords:** Alcohol and tobacco, alcohol, tobacco, and other drug (ATOD) use, abuse, dependence, addiction care, tobacco dependence, smoking, secondhand smoke, nicotine, nicotine replacement, tobacco dependence screening, tobacco dependence treatment, treatment facility-based prevention, co-treatment, treatment issues, treatment barriers, treatment provider characteristics, treatment staff, staff training, AODD counselor, client counselor interaction, smoking cessation, Tobacco Dependence Program at the University of Medicine and Dentistry of New Jersey

## Abstract

Despite the high prevalence of tobacco use among people with substance use disorders, tobacco dependence is often overlooked in addiction treatment programs. Several studies and a meta-analytic review have concluded that patients who receive tobacco dependence treatment during addiction treatment have better overall substance abuse treatment outcomes compared with those who do not. Barriers that contribute to the lack of attention given to this important problem include staff attitudes about and use of tobacco, lack of adequate staff training to address tobacco use, unfounded fears among treatment staff and administration regarding tobacco policies, and limited tobacco dependence treatment resources. Specific clinical-, program-, and system-level changes are recommended to fully address the problem of tobacco use among alcohol and other drug abuse patients.

Tobacco dependence is one of the most common substance use disorders and a leading cause of morbidity and mortality in addiction treatment programs (American Psychiatric Association [APA] 2006; Ziedonis and Williams 2003). Not surprisingly, people who successfully maintain abstinence from alcohol and other drugs often will prematurely die from tobacco-caused diseases such as coronary artery disease, chronic obstructive pulmonary disease, lung cancer, etc. (Hurt et al. 1996). Multiple biological, psychological, and social factors account for the high co-occurrence of alcohol and tobacco dependence, including genetic evidence showing that people with genetic vulnerability to one disorder also are vulnerable to the other. The common genetic vulnerability may be located on chromosome 2 (Bierut et al. 2004; True et al. 1999; See also the article by Grucza and Bierut in this issue).

Despite the existence of effective, evidence-based nicotine dependence treatments, tobacco dependence is commonly ignored in addiction treatment programs. Why has tobacco dependence treatment not been routinely integrated into addiction treatment programs? What are the barriers? Interestingly, 100 years ago the treatment of alcohol, opiates, and cocaine problems included treating tobacco dependence (Hoffman and Slade 1993). Addiction was perceived as a unitary problem, and the use of either the primary substance or any other substance use was considered a potential trigger for the primary addiction. What led to the decision to defer to primary care for the treatment of tobacco dependence? This paper will attempt to answer these questions and to make recommendations for addressing tobacco use in addiction treatment programs.

## Integrating Tobacco Dependence Treatment at the Clinical, Program, and System Levels

Addiction treatment professionals eventually recognized that co-occurring mental illness and addiction needed to be addressed in the context of addiction treatment programs. Likewise, members of this field are beginning to recognize that there also is a need to integrate tobacco dependence treatment across the continuum of substance abuse treatment and prevention services. Improved health services interventions for tobacco dependence are needed at the clinical, program, and system levels (Stuyt et al. 2003). Clinical-level change requires better screening and assessment of nicotine dependence and the inclusion of tobacco dependence in the treatment plan. Staff training is necessary and an important first step to address attitudes, skills, and knowledge. Staff who traditionally treated “alcohol dependence only” have adapted their skills and knowledge to treat other co-occurring substance use disorders such as marijuana or cocaine addiction. A similar transformation could occur for co-occurring alcohol and tobacco dependence. In addition to staff training, other program-level interventions include developing models that integrate the treatment of alcohol and tobacco dependence, staff training on assessing and treating tobacco dependence, and continuous quality improvement on this topic. Broader system-level interventions include increasing collaboration between health and behavioral health providers, developing policy changes to promote addressing tobacco, and providing financial support for tobacco dependence treatment.

This article reviews commonly perceived barriers to addressing tobacco and health services interventions that can help addiction treatment programs better recognize and treat tobacco dependence.

## Barriers

In addition to program culture and financial barriers to treating tobacco dependence, staff attitudes, skills, and knowledge all influence the lack of attention given to tobacco in addiction treatment programs. Staff attitudes set the tone as to whether tobacco dependence will be addressed; tobacco-dependent staff often are the most resistant to change (Bobo and Davis 1993; Asher et al. 2003; Williams et al. 2005; Hurt et al. 1995). Treatment wisdom discourages major life changes during early recovery for fear of relapse, and the treatment culture has accepted that “quitting tobacco” would be a major life change––although quitting other substances simultaneously is not (Sussman 2002; Joseph et al. 2002).

### Staff Attitudes and Tobacco Use

About 30 to 40 percent of addiction treatment staff in community-based programs are tobacco dependent (Bernstein and Stoduto 1999) compared with about 60 to 95 percent of patients (APA 2006; Lasser et al. 2000; Richter et al. 2004); 22 percent of the general population (Centers for Disease Control and Prevention [CDC] 2005); and 3 to 5 percent of physicians, dentists, and dental hygienists (Goldstein et al. 1998; APA 2006). Staff members who smoke most likely are not going to try to help a patient quit smoking––sometimes as a result of their own guilt and shame about their own smoking. In the authors’ clinical work and role as trainers to other professionals, staff members who smoke often support smoking with their patients as their way to promote a better “therapeutic alliance.” Although spending nontreatment time with patients can be positive (such as taking walks, sharing meals, etc.), engaging in addictive behaviors with a patient is inappropriate and unhelpful for recovery. Smoking with patients also normalizes tobacco addiction and even enhances its value as a therapeutic event. An early first step in program change can include policies to restrict staff smoking with patients. This policy change promotes the addiction professional’s role in promoting health and recovery instead of reinforcing the use of substances to manage feelings and cope with stress.

Providing tobacco-dependent staff with the resources, support, and encouragement for their own tobacco dependence treatment is important for their health, their family’s health, and the patient’s health. In addition, employers have recognized the value of having nonsmoking staff. Health care costs are 40 percent higher for smokers than nonsmokers. In addition, employees who smoke spend about 18 days a year on smoking breaks, cost a company drug plan about twice as much, and are absent from work 26 percent more often than nonsmokers (Tobacco Free Oregon 2003). More employers are recognizing that tobacco use in the workforce reduces productivity and increases costs, and, as a result, some employers have changed their hiring practices and policies regarding employee smoking. More employers are helping staff who smoke to quit but also are only hiring nonsmoking staff.

### Lack of Training

Staff members in addiction treatment settings often receive little or no training in treating tobacco dependence. Fortunately, addiction counselors know how to treat other addictions, and the learning curve is quick and often very rewarding when applied to tobacco. The lack of training for most staff members reflects the field’s blinders to this topic––but this is changing. The National Association of Alcoholism and Drug Abuse Counselors (NAADAC) now offers a Tobacco Addiction Specialist credentialing program and has a policy that “advocates and supports the development of policies and programs that promote the prevention and treatment of nicotine dependence on par with alcoholism and drug dependence” (NAADAC 2006). Some States offer “Tobacco Dependence Specialist Added Qualifications” to their certified alcohol and drug counselor certification programs, and many States now require mandatory tobacco training as part of both initial and recertification for credentialed addiction counselors. Several national training centers provide intensive face-to-face or online training and other resources (see the Textbox on page 230).

**Tobacco Dependence Training Resources for Addiction Treatment Staff**

**University of Massachusetts Medical School, Center for Tobacco Prevention and Control**

The Center is actively engaged in a wide range of tobacco-related research, clinical treatment services, technical assistance, and professional education programs. Available at: www.umassmed.edu/behavmed/tobacco

**University of Medicine and Dentistry of New Jersey (UMDNJ) Tobacco Dependence Program**

The UMDNJ provides training programs on tobacco dependence (including a 5-day comprehensive training program) as well as program consultation, technical assistance, research, and clinical service. Resources on their Web site include information on how to obtain their “Drug Free is Nicotine Free” manual for program change. Available at: www.tobaccoprogram.org

**Mayo Clinic Nicotine Dependence Center Education Program**

This is an intensive, 5-day course focusing on the skills needed to effectively treat tobacco dependence. Available at: http://mayoresearch.mayo.edu/mayo/research/ndc_education/tts_certification.cfm

**Association for the Treatment of Tobacco Use and Dependence (ATTUD)**

The ATTUD is dedicated to the promotion of and increased access to evidence-based tobacco treatment and has developed standards for competencies for tobacco treatment specialists. Many of the national training centers follow these competencies. The ATTUD standards are available at: www.attud.org

**ACT Center for Tobacco Treatment, Education, and Research of the University of Mississippi**

The ACT Center, a program of the University of Mississippi Medical Center School of Dentistry, provides training, education, treatment, and research, including a comprehensive tobacco treatment specialist training program. Available at: http://actcenter.umc.edu/

**University of Wisconsin Medical School, Center for Tobacco Research and Intervention (UW-CTRI)**

The UW-CTRI program provides extensive training and technical assistance to help put tobacco cessation research into practice. Available at: www.ctri.wisc.edu and www.medscape.com/viewprogram/3607 (CME online)

**The Addiction Technology Transfer Center of New England**

Funded by the Substance Abuse and Mental Health Services Administration, Center for Substance Abuse Treatment, the Addiction Technology Transfer Center of New England is offering an online course on the cessation of tobacco use: “Tobacco Cessation Treatment: Best Practices.” Available at: www.attc-ne.org/education/courses/ann262.html

**Center for Substance Abuse Treatment**

*Substance Abuse Treatment for Persons With Co-Occurring Disorders. Treatment Improvement Protocol (TIP) Series 42.* Rockville, MD: Department of Health and Human Services publication no. (SMA) 05-3992. Substance Abuse and Mental Health Services Administration, 2005. This includes several lengthy sections on assessing and treating tobacco dependence. Available at: www.ncbi.nlm.nih.gov/books/bv.fcgi?rid=hstat5.chapter.74073

**U.S. Department of Health and Human Services**

The health consequences of involuntary exposure to tobacco smoke: A report of the Surgeon General, 2006. Available at: www.surgeongeneral.gov/library/secondhandsmoke/

Providing staff training enhances skills and knowledge––and also changes attitudes. With appropriate skills and knowledge, staff members often recognize that part of their role is to treat tobacco dependence––and that this is not just primary care’s responsibility. They can better appreciate that tobacco use must be addressed because of the increased morbidity and mortality among their patients. In addition, environmental tobacco smoke (ETS) affects both smokers and nonsmokers. Children, people with existing cardiac disease, and older adults are particularly vulnerable to the health consequences of ETS. Many staff members are surprised to learn that tobacco use is the number one preventable cause of death in the United States (CDC 2001) (see Figure). More addiction treatment programs and clinicians are recognizing that addressing tobacco dependence is important for promoting wellness and recovery. All smokers should be encouraged to seek tobacco dependence treatment at some point in their recovery, and addiction treatment staff can readily learn to use the evidence-based psychosocial treatments and integrate their use with appropriate medications (including several over-the-counter options). System-level reminders to trigger staff to screen, assess, and treat tobacco dependence routinely are needed to ensure that tobacco dependence treatment skills are utilized (Ziedonis et al. 2006). Strategies for treating patients at all levels of motivation to quit are important. Effective brief interventions for addressing tobacco dependence in less motivated smokers have been evaluated (Steinberg et al. 2004) and may be useful for addressing tobacco in addiction treatment populations.

**Figure:**
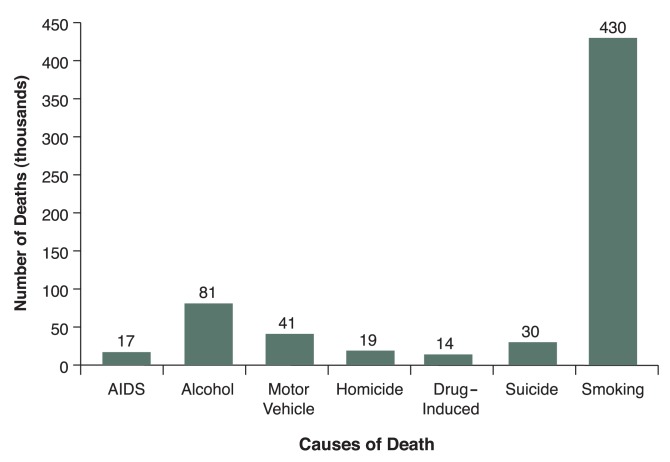
Comparative causes of annual deaths in the United States. Tobacco use is the number one preventable cause of death in the United States. SOURCE: Centers for Disease Control and Prevention (CDC), 2001. Available at: www.cdc.gov/tobacco/research_data/health_consequences/andths.htm

### Clinical Lore

Training staff members to treat tobacco dependence also helps change the treatment culture by correcting many of the misconceptions or “clinical lore” about tobacco––such as “tobacco is not a real drug,” “it’s too hard to address all the substances together,” and “quitting tobacco will definitely worsen other substance recovery.” Clearly, tobacco is both addictive and deadly––even if the serious health consequences are not immediate and do not disrupt the patient’s life as dramatically as other substances with regard to legal, employment, and family problems. Patients are apt to minimize the impact of all their drug use, especially when the consequences are not immediate and visible. Staff members know how to address this type of rationalization and denial regarding other substances. For programs that continue to allow smoking at breaks, there are opportunities to observe patients’ regressive behaviors during breaks––when many behaviors can shift back to a “bar scene.” Staff members can effectively discuss these changes during treatment, as they may mirror prerelapse risk behaviors after discharge.

Some staff members believe that quitting tobacco would be too stressful during treatment. Of course, some patients who smoke will not object, and some patients may also believe that they do not have to stop marijuana when they quit alcohol or stop alcohol use when their primary drug is heroin. Patients may express their own concerns that the urge to smoke will be intolerable, withdrawal will be very difficult, quitting will affect their primary recovery, and they may actually need cigarettes to help them cope with stress (Asher et al. 2003). In fact, evidence suggests that the opposite can occur––that tobacco use can harm, rather than enhance, recovery from other substance use by its ability to trigger other substance use (Williams et al. 2005; APA 2006).

Another potential barrier is that some staff may believe that their patients are just not interested in quitting smoking. As a result, the staff will not discuss the issue of tobacco dependence and quitting. However, many substance abusers are interested in quitting smoking as part of recovery (Ziedonis and Williams 2003; APA 2006). Although more than half of patients who smoke believe that quitting smoking will be the hardest addiction for them to address (Kozlowski et al. 1989), there is evidence that tobacco addiction can be treated successfully in addiction treatment programs, both immediately and later in the recovery process. In a recent meta-analytic review of randomized trials of smoking cessation in substance abuse settings, Prochaska and colleagues (2004) concluded that patients engaging in tobacco dependence treatment had better overall substance abuse treatment outcomes at 6 months after treatment compared with those who did not engage in tobacco dependence treatment. The exact best timing for an individual patient is less clear (Joseph et al. 2004); however, the key is to assess and make a plan to treat tobacco dependence at some point during treatment and/or recovery.

### Smoke-Free Buildings and Resistance to Smoke-Free Grounds

Secondhand tobacco smoke poses a real health risk to everyone exposed to the smoke, and the issue is well addressed in the recent *Surgeon General’s Report on Secondhand Smoke* (U.S. Department of Health and Human Services 2006). The Textbox on page 233 lists the key findings from this report. The need to provide clean indoor air has resulted in policy changes to require smoke-free buildings in many workplaces and public settings, including health care facilities. Although there was initial resistance to smoke-free buildings by some addiction treatment staff, State laws and requirements set by the Joint Commission on Accreditation of Healthcare Organizations have changed the norm to smoke-free buildings for treatment. In addition to smoke-free buildings, some inpatient workplaces (including addiction treatment programs) have taken an additional step toward addressing tobacco by implementing “smoke-free grounds.” This step means that tobacco smoking is not allowed anywhere on the grounds of the addiction treatment program, rather than just being prohibited in the buildings. Having entirely tobacco-free grounds is an additional policy change that some States have now mandated for their treatment programs (see Sidebar on pp. 236–240).

**Steps for Addressing Tobacco Within Addiction Treatment Programs**

1. Acknowledge the challenge to address the barriers and integrate the solutions
2. Establish a leadership group and make a commitment to change
3. Create a change plan and realistic implementation timeline
4. Start with easy program and system changes, including tobacco policies
5. Conduct staff training
6. Assess and document in charts nicotine use, dependence, and prior treatments
7. Incorporate tobacco issues into all patient education curriculums
8. Provide medications for nicotine dependence treatment
9. Provide treatment and recovery assistance for interested nicotinedependent staff
10. Integrate motivation-based treatments throughout the program
11. Establish ongoing communication about system changes with 12-Step recovery groups, professional colleagues, and referral sources
12. Consider additional policies addressing tobacco, including smoke-free grounds

NOTE: Adapted from Order-Connors B. Smoke screen. *Professional Counselor* 11(6):15–52, 1996.

Staff members who smoke often initially oppose the “smoke-free grounds” level of program change. Program leaders, administrators, or staff members also may have concerns that patients will act out, have worse withdrawal, leave against medical advice (AMA), or seek treatment at competing programs that allow smoking. Contrary to expectations, treatment programs with smoke-free grounds often report less acting out, less haggling about smoke breaks/number of cigarettes allowed, less coercion of smokers by either peers or staff, no increase in the AMA discharge rate, increased likelihood of completed treatment, and an increase in the number of patients seeking treatment (APA 2006; Williams et al. 2005; Hurt et al. 1995).

Other cultural milieu barriers are subtle. Some programs still sell cigarettes with the profits contributing to one of the few “discretionary” funds to which these programs have access. The projected loss of these funds obviously contributes to administrative resistance to this change. Some addiction treatment programs are housed within psychiatric care facilities with even less attention to tobacco use.

### Limited Treatment Resources

Available treatment resources––especially coverage for tobacco dependence treatment medications––often are limited for tobacco-dependent staff and patients. This is especially problematic for patients who may have limited income and are underinsured or uninsured. In the general population, psychosocial behavioral therapy alone can be as effective as medications alone in the treatment of tobacco dependence (APA 2006). However, there is a much greater likelihood of receiving only medications for tobacco dependence treatment. Integrating psychosocial tobacco dependence treatment into addictions treatment is an effective way to overcome some of the financial issues. For example, psychosocial treatment interventions in addiction treatment programs commonly address multiple drugs for any individual because other drugs (including tobacco) are triggers for the primary addiction. Integrating smoking cessation into routine addiction psychosocial treatment helps the primary addiction and does not require additional billing specific to tobacco dependence to the insurance company. As with other multiple addictions, charges for psychosocial treatment are bundled so that programs address multiple problems under the primary substance use disorder. Many inpatient programs either do not have tobacco dependence treatment medications on their pharmacy formulary or the options are very limited. Outpatient programs are more reliant on the patient’s health care benefits or willingness to pay out of pocket for these medications. Although the cost of over-the-counter nicotine replacement still is less than the cost of a carton of cigarettes, most patients still perceive that this out-of-pocket cost is too high and feel entitled to benefits covering those costs––even if they are not covered.

**Surgeon General’s Summary of the Effect of Secondhand Smoke**

1. Secondhand smoke causes premature death and disease in children and in adults who do not smoke.
2. Children exposed to secondhand smoke are at an increased risk for sudden infant death syndrome (SIDS), acute respiratory infections, ear problems, and more severe asthma. Smoking by parents causes respiratory symptoms and slows lung growth in their children.
3. Exposure of adults to secondhand smoke has immediate adverse effects on the cardiovascular system and causes coronary heart disease and lung cancer.
4. The scientific evidence indicates that there is no risk-free level of exposure to secondhand smoke.
5. Millions of Americans, both children and adults, are still exposed to secondhand smoke in their homes and workplaces despite substantial progress in tobacco control.
6. Eliminating smoking in indoor spaces fully protects nonsmokers from exposure to secondhand smoke. Separating smokers from nonsmokers, cleaning the air, and ventilating buildings cannot eliminate exposures of nonsmokers to secondhand smoke.

SOURCE: U.S. Department of Health and Human Services. *The Health Consequences of Involuntary Exposure to Tobacco Smoke: A Report of the Surgeon General*. Atlanta, GA: U.S. Department of Health and Human Services, Centers for Disease Control and Prevention, Coordinating Center for Health Promotion, National Center for Chronic Disease Prevention and Health Promotion, Office on Smoking and Health, 2006.

## Solutions

Over the past 10 years, many addiction treatment agencies have begun to better address tobacco dependence and have benefited from program-level interventions (Stuyt et al. 2003). One organization doing these health services interventions—the University of Medicine and Dentistry of New Jersey (UMDNJ) Tobacco Dependence Program—has helped many addiction treatment programs incorporate evidence-based tobacco dependence treatment into ongoing practice. In some cases, these programs have adopted a “motivation-based treatment” model to address tobacco dependence, which does not require abstinence by the patient, but all patients who are tobacco dependent get screened, assessed, and offered some type of treatment.

Although addiction treatment programs use urine toxicology screens and breathalyzers to screen for alcohol and other drugs, most do not screen for tobacco use with a carbon monoxide (CO) meter. The CO meter is a good measure of tobacco smoking exposure and can be used as an effective tool to motivate patients to seek tobacco dependence treatment (Steinberg et al. 2004).

Education and other motivational enhancement interventions can help less motivated patients to incrementally increase their commitment to quit. For example, information about health risks, wellness interventions (stress management, nutrition, and exercise), Stage II Recovery, available medication and other treatments, local and online Nicotine Anonymous meetings, and other community treatment resources (e.g., State-supported Internet sites and telephone quit lines) can immediately help motivate some individuals. Others may save this information for a later quit attempt. More motivated patients can aim for tobacco abstinence and be effectively treated when psychosocial and medication treatments are blended into the “treatment as usual.” Program-level interventions include staff training, policy changes, and, in some cases, establishing smoke-free grounds.

An initial health service research study has found that the UMDNJ program intervention can be effective in addressing tobacco dependence at the residential treatment program level, and another more rigorous health services study funded by the National Institute on Drug Abuse (NIDA) currently is underway to study this approach in the context of three community- based treatment programs within the NIDA Clinical Trial Network. The UMDNJ Tobacco Dependence Program co-leads this project and provides consultation and training to the programs. The consultation follows the steps outlined in the Textbox on page 232.

Developing a leadership team with a game plan is a necessary first phase of the program intervention. Through that process the organization’s “motivational level” for addressing tobacco can be better determined. Meaningful change requires local champions of the change process. Resources of time and money are needed. Paradigm shifts are required, and staff training is essential. Because tobacco dependence is insidious in most addiction treatment programs, the leadership team should include representatives from the whole organization (i.e., administration, staff, union, housekeeping, security, grounds, etc.). Some system changes include modifying standard intake forms to include a comprehensive tobacco dependence assessment, including tobacco on the treatment planning forms, providing patient education literature, posting pro-wellness posters and no-smoking signs, and starting local Nicotine Anonymous groups. Other changes can include developing policies specific to tobacco use, labeling smoker’s charts, changing the name of “smoke breaks” to just “breaks,” not allowing staff members to smoke with patients, and providing nicotine replacement therapies or other Food and Drug Administration–approved medication for smokers on the inpatient units and possibly at other levels of care.

When implementing tobacco-related policy changes, it is helpful to ensure that such changes are not solely perceived as losses (e.g., we have all just lost our right to smoke). It may be helpful to provide a pleasant alternative during the transition. Individual programs should come up with strategies that work for them. One program, for example, replaced smoke breaks with “popcorn breaks,” with the agency providing free popcorn.

Tobacco-dependent patients should have the resources available (including trained staff ) to help them quit, and patients and staff members who do not smoke should not be exposed to the toxins of ETS. There are clear barriers to addressing tobacco use and dependence, but there also are effective ways to address these barriers and promote the integration of evidence-based tobacco dependence treatment into addiction treatment programs.

The addiction treatment community as a whole now has an opportunity to denormalize tobacco use for the field by tailoring traditional tobacco control strategies to the unique issues of the addiction treatment and recovery community. Denormalization of tobacco use includes making smoking behavior not the norm and providing education about the health risks of tobacco products and the activities of the tobacco industry (e.g., Truth Campaign [Thrasher et al 2004]).

Although tobacco control strategies have effectively denormalized tobacco use in the general population (Hammond et al. 2006), these strategies have not targeted people with substance use disorders. Tobacco control efforts within the addiction treatment and recovery community could help the field to recognize and manage tobacco dependence as any other substance use disorder. Denormalization strategies in this setting would include assessing and treating tobacco dependence in treatment programs, maintaining smoke-free buildings and grounds, eliminating the sale and advertisement of tobacco products, improving understanding of the impact of smoking in the home on the children of people in recovery, and perhaps revealing how the tobacco industry may target people with other addictions (many of their ads link alcohol and tobacco). Targeted mass media campaigns have been effective in reducing tobacco use in the general population, and opportunities exist to develop a media campaign for the addiction treatment and recovery community. The leaders of Alcoholics Anonymous, Bill W. and Dr. Bob, were both smokers and died of tobacco-caused diseases before the health consequences and addictive nature of tobacco use were fully recognized. Undoing the “normalization of tobacco” that has occurred within the addiction treatment and 12-Step community for the last generation will need input from everyone involved in the treatment, prevention, and recovery community.

## Conclusion

Tobacco dependence is one of the most common addictions among people with alcohol and other drug addictions––and a leading cause of morbidity and mortality in addiction treatment programs. Now is the time for addiction treatment programs to better address tobacco dependence at the clinical, program, and system levels. Many programs have been successful at doing so. Then there are real and perceived barriers to address, but as with recovery from any substance, the first step is to acknowledge the need for change. There are then many successful ways to begin and support that change.
